# *In vitro* screening for anti-acetylcholiesterase, anti-oxidant, anti-glucosidase, anti-inflammatory and anti-bacterial effect of three traditional medicinal plants

**DOI:** 10.1080/13102818.2014.969877

**Published:** 2014-10-28

**Authors:** Doaa A. Ghareeb, Amani M.D. ElAhwany, Sherif M. El-mallawany, Ashraf A. Saif

**Affiliations:** ^a^ Biochemistry Department, Faculty of Science, Alexandria University, Alexandria, Egypt; ^b^ Plant and Microbiology Department, Faculty of Science, Alexandria University, Alexandria, Egypt; ^c^ Chemistry Department, Faculty of Science, Alexandria University, Alexandria, Egypt; ^d^ Al-Leith University College, Umm Al-Qura University, Makkah, Saudi Arabia

**Keywords:** *Calluna vulgaris*, *Tribulus terrestris*, *Ferula hermonis*, anti-diabetic, Alzheimer treatment

## Abstract

In this study we investigated the phytoconstituents *Calluna vulgaris*, *Ferula hermonis* and *Tribulus terrestris*, and then assessed their possible biological activities by using standard methods. A preliminary phytochemical investigation of the three extracts revealed the presence of alkaloids, flavonoids, proteins, lipids, phenolic compounds, saponins, sterols and amino acids. Three extracts showed anti-oxidant effect as they inhibited the 1,1-diphenyl-2-picryl hydrazyl (DPPH) oxidation and production of thiobarbituric acid reactive substances (TBARS). Moreover, three extracts showed anti-acetylcholiesterase (AChE) and this effect was concentration dependent. *C. vulgaris* was the most potent inhibitor of AChE. Furthermore, the three plant extracts had an inhibitory effect toward α-glucosidase. The inhibitory effect was concentration dependent and the most potent inhibitor for α-glucosidase was the extract from *T. terrestris. Calluna vulgaris* showed anti-inflammatory effect at tested concentrations while the other two extracts exhibited this effect only at concentration of 25 μg/mL. Finally, *C. vulgaris* had a significant effect against pathogenic bacteria (*Agrobacterium tumefaciens*, *Erwinia* sp., *Klebsiella pneumonia* and *Pseudomonas aeruginosa*) in comparison to other extracts from *Ferula* sp., or *Tribulus* sp. In conclusion, all tested extracts could be promising sources for the treatment of diabetes, Alzheimer's disease, infectious diseases and oxidative stress related disorders because they are rich in phenols and flavonoids that give anti-oxidant molecules and produce an inhibitory effect against the tested enzymes.

## Introduction

Green Chemistry is the design, development and implementation of chemical products and processes to decrease or eliminate the use and production of substances that are harmful to human health and the environment. Green Chemistry is a basic fundamental way to implement sustainable development.

The application of the Twelve Principles of Green Chemistry has proved that it is possible to arrive at a compromise between society and economy, resources and environment, equity and efficiency, mankind and nature by designing sustainability at the molecular level.

There are 12 fundamental principles of Green Chemistry, and according to the 7th, the use of renewable raw materials is of great importance to Green Chemistry practice and there is growing urgency to develop bio-based products produced from renewable resource. Bio-derived material is made from renewable agricultural and forestry feed stocks including wood, wood waste and residues, grasses, crops and crop by-products.[1]

Heather, *Calluna vulgaris*, is an excellent feedstock for natural extracts with a wide range of biological activities. It is a small evergreen shrub, native to Europe but introduced also to Atlantic Northern America and to New Zealand. It is a dominating species in health and common in Northwest Europe but can be found from Spain to Scandinavia and from the Azores to the Ural Mountains. Heather serves as a food source for several mammals, reptiles and insects. People have also used heather for thatching, making ropes, bedding, brooms and ale. Traditionally, its flowers and herbs are used to treat urinary tract disorders, and as an antiseptic, wound healing, antirheumatic, expectorant and choleretic remedy. Pharmacological studies showed anti-inflammatory, anti-oxidant, antiproliferative and monoamine oxidase-A (MAO-A) inhibitory effects.[2] Its seeds showed moderate anti-bacterial activity. Herbs and flowers of *C. vulgaris* are still widely collected from the wild. Their high phenolic content and the biological activity of these compounds serve as the basis for its beneficial effect as a medicinal plant.[3]

The extract from the flowers of the plants contains phytochemicals such as carotenoids and flavonoids, which are beneficial for protection of the skin from free radicals. The astringent property of the plant makes the extract a great additive for soaps and facial cleansers. For example, ABS Heather Extract G is produced from Irish moss. Furthermore, both *in vitro* and *in vivo* studies revealed anti-oxidant and anti-inflammatory effects of *C. vulgaris* extract. *In vitro* and *in vivo* studies demonstrated that kaempferol and quercetin, the most important compounds identified in *C. vulgaris*, exhibit valuable anti-oxidant, anti-inflammatory and antiproliferative activities.[4]

The genus *Ferula* (Apiaceae), known as a good source of biologically active compounds, comprises about 170 species widely distributed throughout the Mediterranean area and Central Asia. Several species of the genus *Ferula* have been used in traditional medicine for a variety of therapeutic purposes such as tranquilizers, and for treatment of digestive disorders, rheumatism, headache, arthritis, dizziness, toothache, etc. *Ferula hermonis* Boiss., commonly known as ‘Shilsh-el-zallouh’ or ‘Hashishat-al-kattira’, is a small shrub that grows abundantly on the Hermon Mountain between Syria and Lebanon. This plant has long been used in the Middle East as an aphrodisiac, and for the treatment of frigidity and impotence. Furthermore, several studies were conducted to estimate the hormonal activity of the isolated sesquiterpenes from *F. hermonis* and recently herbal products containing *F. hermonis* extracts have been sold at the dietary supplement market claiming a sexual function enhancement effect. Different activities were also reported including anti-inflammatory, cytotoxic and other effects.[5]

*Tribulus terrestris* L. (Zygophyllaceae), also called ‘Puncture vine’, is a prostrate annual herb native to the Mediterranean region, but widely distributed in warm regions of Europe, Asia, America, Africa and Australia. It is about 30–70 cm high, grows as a summer annual plant, with pinnately leaves, yellow flowers and stellate shaped carpel fruits. These plant parts are known to be used in the traditional herbal medicine for treatment of various ailments such as kidney infection, impotence, cancer and the herb's fruits possess antihypertensive activity. The herb has been used as tonic, aphrodisiac, astringent, analgesic, stomachic, anti-hypertensive, diuretic and urinary anti-septic and treatment for sexual and erectile dysfunctions. Furthermore, its extract is also commonly used among the folk medicine tradition for control of blood pressure and cholesterol. There are reports showing that this extract decreases blood cholesterol levels in humans, rats and mice. However, data concerning the effect of *T. terrestris* extract on poultry are not present.[6]

One of the uses of *T. terrestris* is in urinary infections. The ethanolic extract of Yemeni *T. terrestris* has demonstrated no detectable anti-bacterial activity against any of the reference bacteria. However, the ethanolic extracts of all parts (fruits, stems plus leaves and roots) of Turkish *T. terrestris* showed activity against all reference bacteria. Moreover, ethanolic extracts of the fruit and leaf of Indian *T. terrestris* were active against *Escherichia coli* and *Staphylococcus aureus*.[7]

The medicinal values of plants lie in their phytochemical components which produce definite physiological actions on the human body. The most important of these components are alkaloids, tannins, flavonoid and phenolic compounds. Phytochemicals are extensively found at different levels in various medicinal plants and used in herbal medicine to treat diverse ailments such as cough, malaria, wounds, toothache and rheumatic diseases, diabetes, inflammation, Alzheimer disease, disease related to reactive oxygen species (ROS) formation and bacterial infection.

The majority of disease/disorders are mainly linked to oxidative stress due to the presence of ROS. The most common ROS are superoxide anion (O2˙¯), hydroxyl radicals (OH˙) and hydrogen peroxide (H_2_O_2_) which has been implicated in the etiology and pathophysiology of human diseases such as inflammation, viral infections, autoimmune pathologies and ulcer. ROS can readily react with and oxidize most bio-molecules including carbohydrates, proteins, lipids and DNA. Recently, there is an increasing interest in finding natural anti-oxidants from plant materials to replace synthetic ones. Natural anti-oxidant compounds which are widely distributed in plants are capable of terminating a free radical-mediated oxidative reaction and would have beneficial activities in protecting the human body from such diseases. The ability of phenolic compounds to serve as anti-oxidants has been recognized by donating a hydrogen atom.[8]

Inflammation is a normal protective response to tissue injury and it involves a complex array of enzyme activation, mediator release, fluid extravasations, cell migration, tissue breakdown and repair. It is a complex process, which is frequently associated with pain and involves occurrences such as increase in vascular permeability, increase of protein denaturation and membrane alterations. It is believed that the currently available drugs such as opioids and non-steroidal anti-inflammatory drugs (NSAIDs) are not useful in all cases of inflammatory disorders, because of their side effects and potency.[9] Therefore, search for other alternatives seems necessary and beneficial.

Common drugs approved for the therapy of Alzheimer's disease (AD) act by counteracting the acetylcholine deficit, and try to enhance the levels of acetylcholine in the brain. Acetylcholine is involved in the signal transfer in the synapses. After being delivered in the synapses, acetylcholine is hydrolysed giving choline and acetyl group in a reaction catalysed by the enzyme acetylcholinesterase. The molecular basis of the Alzheimer drugs used so far takes advantage of their action as acetylcholinesterase inhibitors. Some of the drugs approved for therapeutic use show hepatotoxicity; consequently, there have been a continuous search for new medications.[10]

The early stage of diabetes mellitus type 2 is associated with post-prandial hyperglycaemia due to impaired acute insulin secretion following meals. Hyperglycaemia is believed to increase the production of free radicals and ROS, leading to oxidative tissue damage and diabetic complications such as nephropathy, neuropathy, retinopathy and memory impairment. Glucosidases are a group of digestive enzymes which break down the dietary carbohydrates into simple monosaccharide. Glucosidase inhibitors such as acarbose reduce the rate of carbohydrate digestion and delay the carbohydrate absorption from the digestive tract. Therefore, they have a potential to prevent the development of type 2 diabetes mellitus by lowering the after-meal glucose levels.[11]

The use of and search for drugs and dietary supplements derived from plants have accelerated in recent years. Ethnopharmacologists, botanists, microbiologists and natural-products chemists are combing the Earth for phytochemicals and ‘leads’ which could be developed for treatment of infectious diseases. Plants are rich in a wide variety of secondary metabolites, such as tannins, terpenoids, alkaloids and flavonoids, which have been found *in vitro* to exhibit antimicrobial properties.[12]

Infectious diseases are caused by pathogenic microorganisms, such as bacteria, viruses, parasites or fungi. Diseases can spread, directly or indirectly, from one person to another. Infectious diseases are the second leading cause of death worldwide. About one-fourth of all the medicines we use come from rainforest plants. However, scientific studies have been conducted only to a limited extent with few medicinal plants. The development of bacterial resistance to presently available antibiotics has necessitated the search of new anti-bacterial agents. In rural and backward area of India, several plants are commonly used as herbal medicine for the treatment of infectious diseases. Four such plants commonly used by the people of the area were screened for potential anti-bacterial activity.[13]

The aim of this study was to explore the phytochemical constituents of *C. vulgaris*, *T. terrestris* and *F. hermonis*, to bioscreen their crude extracted bioactive ingredients and to show the anti-bacterial properties against some microorganisms.

## Materials and methods

### Plant materials and chemicals

Plants were obtained from the local market and authenticated by Prof. Salma Eldareir from the Botany Department of Alexandria University, Egypt. The local and international herbarium for these plants were used as a guide for plants’ definition such as voucher No. 2008FH, at the Pharmacognosy Department, Assiut University, Assiut, Egypt for *F. hermonis*; 02-444 Czech: Francensbad, August 1880 and Robert W. Freckmann Herbarium (http://wisplants.uwsp.edu/scripts/detail.asp?SpCode=CALVUL) for *C. vulgaris* (L.); and finally, voucher No (Minia-04-Mar-TT) Faculty of Pharmacy, Minia University, Egypt for *T. terrestris*.

Trichloroacetic acid (TCA), thiobarbituric acid (TBA), berberine chloride, butylated hydroxytoluen (BHT), acetylthiocholine iodide (ACTI), 1,1-diphenyl-2-picryl hydrazyl (DPPH) radical, p-nitrophenyl-β-D-glucopyranoside (PNPG), 5,5’-dithiobis 2-nitrobenzoic acid (DTNB) and sodium nitroprusside were purchased from Sigma Chemical Co. (St. Louis, Mo, USA). Organic solvents of HPLC-grade like ethanol 95%, methanol and petroleum ether were obtained from Merck (USA). All other chemicals and reagents were of analytical grade.

### Qualitative phytochemical screening

Dried powdered plant roots were phytochemically screened for alkaloids, saponins, flavonoids, amino acids, and total content of carbohydrates, proteins, phenol and lipids.[14]

### Extraction of the plants’ materials

The dried powdery roots (100 g) of the plants were exhaustively defatted with petroleum ether and subjected to steam distillation method for ethanolic gradient extraction with Soxhlet apparatus (300 mL ethanol was used for each plant). The ethanolic extract was concentrated to minimum volume using rotary evaporator (Buchi, Switzerland) then lyophilized (DISHI, DS-FD-SH10, Xi’an Heb Biotechnology Co, China) to obtain a powder extract of the tested plants. *Calluna vulgaris*, *T. terrestris* and *F. hermonis* gave 25, 20 and 15 g crude extracts, respectively. Our plant extract was dissolved at concentration of 1 mg/mL 50% ethanol.

### Quantitative determination of phytoingredients in the tested plant extracts

The method mentioned by Shamsa et al. [15] was carried out to estimate the amount of alkaloid in the tested plants. For saponins, the method described by Hiai et al. [16] was used. Total phenolic constituents of plant extracts were performed employing the literature methods involving the Folin–Ciocalteu reagent and gallic acid as standard.[17] While the total flavonoid content was determined according to the aluminium chloride colourimetric method described by Chang et al. [18]. On one hand, total protein content was determined according to the method described by Lowry et al.[19] On the other hand, the amino acid content was determined by the ninhydrin method described by Jones et al.[20] Finally, total lipid and carbohydrate levels were determined, respectively, according to Zollner and Kirsch [21] and applying the anthrone method.[22]

### *In vitro* induction of lipid peroxidation in biological systems

*In vitro* lipid peroxidation was induced in oleic acid by a method as described by El-Sayed et al. [23] with some modifications. The following biological screening assays were used.

### Lipid peroxidation prevention

A total of 0.5 mL of plant extract (1 mg/mL) (test & blank), the organic solvent (positive control & test & blank) or distilled water (dH_2_O) (blank & negative control) was incubated with an equal volume of oleic acid for about 45 min at 37 °C except the blank. *In vitro* lipid peroxidation was induced by adding H_2_O_2_ and ferrous sulphate (FeSO_4_·7H_2_O) at a final concentration of 1 and 0.5 mM, respectively, in both test and control reaction mixtures. After an incubation period of about 45 min at 37 °C, BHT at a final concentration of 0.02% was added and mixed carefully to stop the peroxidation reaction. The mixtures were centrifuged at 3000 rpm for 15 min, then 1 mL of the resultant supernatant was mixed with 1 mL of TCA (15%) followed by centrifugation at 3000 rpm for 10 min, then 1 mL supernatant was mixed with 0.5 mL TBA (0.7%) followed by BWB and then the absorbance was read at 532 nm against blank.

### DPPH radical-scavenging activity

The stable DPPH radical was used for determination of free radical-scavenging activity of the extracts.[24] Different concentrations of each extracts were added, at an equal volume, to methanolic solution of DPPH (100 μM). After 15 min at room temperature, the absorbance was recorded at 517 nm. The experiment was repeated three times. Vitamin C was used as standard controls. IC_50_ values denote the concentration of sample, which is required to scavenge 50% of DPPH-free radicals.

### *In vitro* anti-inflammatory activity

*In vitro* anti-inflammatory activity of extracts was assessed by the human red blood corpuscles (HRBCs) membrane stabilizing method [25] with slight modifications. The blood was collected from healthy human volunteer who had not taken any anti-inflammatory drugs for two weeks prior to the experiment, transferred to heparinized centrifuge tubes and centrifuged at 3000 rpm. The packed cells were washed with isosaline and a 10% suspension in normal saline was made. Diclofenac potassium (50 mcg/mL) was used as a standard. The reaction mixture (4–5 mL) consisted 2 mL of hypotonic saline (0.25% w/v NaCl), 1 mL of 0.15 M phosphate buffer (pH 7.4), one mL of test solution (1 mg/mL) in normal saline and 0.5 mL of 10% HRBC in normal saline. For control, 1 mL of isotonic saline was used instead of test solution. The mixtures were incubated at 56 ºC for 30 min, cooled at running tap water, and centrifuged at 3000 rpm for 20 min. The absorbance of supernatant was read at 560 nm using a visible spectrophotometer. The experiment was performed in triplicates. The control represents 100% lyses. The percentage membrane stabilization was calculated by using the following formula:% inhibition of haemolysis = 100 × [absorbance of control – absorbance of test]/absorbance of control.

### Assay of nitric-oxide-scavenging activity

The procedure is based on the principle that sodium nitroprusside in an aqueous solution at physiological pH spontaneously generates nitric oxide which interacts with oxygen to produce nitrite ions which can be estimated using Griess reagent. Scavengers of nitric oxide compete with oxygen, leading to reduced production of nitrite ions. For the experiment, sodium nitroprusside (10 mM), in phosphate-buffered saline, was mixed with different concentrations of each extract dissolved in water and incubated at room temperature for 150 min. After the incubation period, 0.5 mL of Griess reagent was added. The absorbance of the formed chromophore was read at 546 nm. Quercetin was used as a positive control.[26]

### *In vitro* cholinergic effect

Acetylcholiesterase (AChE) activity was measured according to the method of Ellman et al. [27]. Phosphate buffer with a volume of 130 μL (0.1 M pH 7.4) was added to a mixture of 20 μL of purified AChE enzyme and 20 μL of plant extract (test) or organic solvent (control), and then incubated for 45 min at 37 °C. Then, 5 μL of substrate ACTI (75 mM) was added, mixed well and incubated for 15 min at 37 °C. Following this, 60 μL of DTNB (0.32 mM) was added and left for 5 min. The absorbance was measured at 405 nm and the specific activity was calculated.

### *In vitro* anti-diabetic/diabetic effect

The method mentioned by Han and Srinivasan [28] was carried out with a slight modification to estimate the effect of the plant extracts on the activity of α-glucosidase (EC 3.2.1.20). An aliquot of 100 μL from each plant extract (1 mg/mL) (test), organic solvents (control) or dH2O (blank) was diluted with 2.5 mL 0.1 M phosphate buffer with pH 7.4. An equal amount (100 μL) of purified enzyme was added, mixed well and incubated in a water bath with the reaction mixture at 30 °C for 5 min. Next, 500 μL PNPG (5 mM) was added and the reaction was allowed to proceed for 15 min. The reaction was then stopped by the addition of 2 mL of 1 M Na_2_CO_3_. The produced colour was spectrophotometrically detected at 400 nm. A unit of enzyme activity was defined as the released *n* moles of p-nitrophenol/min.

### Determination of anti-bacterial effect

The used microorganisms for the antimicrobial activity assay were *S. aureus*, *Klebsiella pneumonia* and *Pseudomonas aeruginosa* obtained from the Medical Research Centre, while the plant pathogens *Erwinia* sp., and *Agrobacterium tumefaciens* were obtained from the Department of Plant Pathology, Faculty of Agriculture, Alexandria. These were all cultured in an LB broth at 37 °C and maintained on LB agar (Luria Bertani; in g/l: Tryptone, 5; Yeast extract, 5; NaCl, 10; sterilized by autoclaving for 25 min at 121 °C). The pH was adjusted to 7 with 1 N NaOH or 1 N HCl prior to sterilization. After sterilization media were supplemented with 175 mg/mL mycostatin to inhibit fungal growth. All cultures were supplemented with 15% glycerol (w/v) and stored frozen at 4 °C.

The assay was performed as previously described by Senthil Kumar and Kamaraj [29]. Each pathogen was grown on its medium of isolation and incubated at 30 °C until visible growth. Bacterial suspension of each indicator pathogen was then plated with the LB media in the agar plates. A well was cut in the middle of the plate and 500 μL of extract was added in it. The plate was incubated at 30 °C for 24–48 h. Inhibition zones were scored as anti-bacterial activity measured in cm.

### Statistical analysis

The values were represented as mean ± SEM (standard error of **mean**) and the data obtained from this study were subjected to a one-way analysis of variance (ANOVA) followed by Student's *t*-test. The values of *p* < 0.05 were considered to indicate the levels of significance.

## Results and discussion

The preliminary phytochemical investigation of the three extracts revealed the presence of various secondary metabolites such as alkaloids, flavonoids, proteins, lipids, tannins, phenolic compounds, saponins, sterols and amino acids but these particular extracts did not contain carbohydrates (Table 1). Data represented in Table 1 also showed that the extract from *C. vulgaris* had the highest content of total phenolic, flavonoids and proteins. Furthermore, the extract from *F. hermonis* showed the highest alkaloid and total lipid content. Finally, the *T. terrestris* extract had the highest flavonoid, saponins and amino acid composition. In agreement with our results, it is reported that *T. terrestris* methanolic extract had several phytoingredients such as spirosaponins. Phytochemical studies of *C. vulgaris* have shown the presence of phenolic components such as flavonoid glycoside and kaempferol-3-*O*-β-d-galactoside.[30] Other constituents of heather plants are various types of quercetin, tannins, flavonoids [31], arbutin, hydroquinone, glycosids, saponins and mineral compounds. Isolation of chromones and triterpenes from the plant has also been reported.[2] Finally, *F. hermonis* extracts mainly contained aromatic esters of sesquiterpene alcohol known as ferutinin (ferutinol p-hydroxybenzoate), teferdin (ferutinol benzoate), teferin (ferutinol vanillate) and epoxybenz (epoxyferutinol benzoate). In addition to these esters, several naturally occurring vitamins and minerals were found in the roots of *F. hermonis*.[32]

**Table 1.** Quantitative phytochemical screening of the plants.

Components	Plant	% concentration ± SD
Alkaloid	*Calluna vulgaris*	0.0131 ± 0.0024
	*Tribulus terrestris*	0.0176 ± 0.0056
	*Ferula harmonis*	0.0483 ± 0.0040
Total phenol	*Calluna vulgaris*	83.229 ± 6.855
	*Tribulus terrestris*	30.104 ± 1.573
	*Ferula harmonis*	26.563 ± 2.742
Flavonoid	*Calluna vulgaris*	44.849 ± 5.915
	*Tribulus terrestris*	45.152 ± 6.701
	*Ferula harmonis*	8.485 ± 2.922
Total protein	*Calluna vulgaris*	447.88 ± 355.38
	*Tribulus terrestris*	362.88 ± 446.63
	*Ferula harmonis*	104.13 ± 196.63
Total lipid	*Calluna vulgaris*	1.043 ± 0.109
	*Tribulus terrestris*	0.996 ± 0.091
	*Ferula harmonis*	1.063 ± 0.070
Saponin	*Calluna vulgaris*	2542.86 ± 363.66
	*Tribulus terrestris*	9171.43 ± 525.28
	*Ferula harmonis*	3242.86 ± 60.61
Amino acid	*Calluna vulgaris*	279 ± 65.997
	*Tribulus terrestris*	962.33 ± 447.83
	*Ferula harmonis*	329 ± 18.856
Total carbohydrate	*Calluna vulgaris*	ND
	*Tribulus terrestris*	ND
	*Ferula harmonis*	ND

Note: ND: not detected.

Phenols and polyphenolic compounds, such as flavonoids, are widely found in food products derived from plant sources, and they have been shown to possess significant anti-oxidant properties. The high amount of phenols and flavonoids in extracts may explain their high anti-oxidative activities.[33] The three plant extracts had anti-oxidant properties as they inhibited the formation of thiobarbituric acid reactive substances (TBARS) and oxidation of DPPH in a concentration-dependent manner which is also shown in Table 2. The extract from *C. vulgaris* exhibited the highest anti-oxidant activity with IC_50_ of 40.8 ± 2.3 μg/mL followed by the *F. hermonis* extract with IC_50_ of 90.9 ± 1.5 μg/mL and finally IC_50_ for the *T. terrestris* extract was 492.3 ± 2.9 μg/mL. The model of scavenging the stable DPPH radical is a widely used method to evaluate the free radical-scavenging ability of various samples. DPPH is a stable nitrogen-centred free radical which colour changes from violet to yellow upon reduction by either the process of hydrogen or electron donation. Substances which are able to perform this reaction can be considered as anti-oxidants and therefore radical scavengers.[34] This activity may be due to the presence of phenolic compounds in the tested extracts. Generally, the reducing properties are associated with the presence of compounds which exert their action by breaking the free radical chain through donation of a hydrogen atom. The ability of these compounds to give hydrogen atoms may be due to their polyphenolic nature. Ibraheim et al. [5] proved that daucane sesquiterpenes from *F. hermonis* Boiss acted as anti-oxidants as they inhibited DPPH oxidation. It is known that phenolic compounds are plant secondary metabolites with anti-oxidative, anti-inflammatory and photoprotective properties. *In vitro* and *in vivo* studies demonstrated that kaempferol and quercetin, the most important compounds identified in the extract from *C. vulgaris*, have important anti-oxidant, anti-inflammatory and antiproliferative properties.[35]

**Table 2.** Anti-oxidant effect of the tested extracts.

	*Calluna vulgaris*		*Ferula hermonis*		*Tribulus terrestris*	
Concentration	TBARS inhibition (%)	DPPH scavenging (%)	TBARS inhibition (%)	DPPH scavenging (%)	TBARS inhibition (%)	DPPH scavenging (%)
25 ug/mL	20 ± 2.1a	30 ± 1.1a	11 ± 0.9a	21 ± 4.3a	18 ± 1.4a	23 ± 5.1a
50 ug/mL	30 ± 1.9b	45 ± 3.1b	29 ± 2.1b	32 ± 1.7b	29 ± 3.2b	41 ± 3.9b
70 ug/mL	51 ± 3.5c	60 ± 5.1c	41 ± 5.6c	45 ± 2.9c	33 ± 2.6b	51 ± 3.2c
100 ug/mL	64.2 ± 4.2d	80 ± 3.5d	57.8 ± 3.4d	69 ± 5.3d	43 ± 2.8c	54 ± 1.2c

Within the column, means with different letters (a, b, c, or d ) were significantly different at *p* < 0.05. Mean with letter (a) was significantly the lowest value while mean with the letter (d) was significantly the highest value. If two or three groups have the same letters that means there is no significant difference detected at *p* < 0.05.

**Figure 1.**
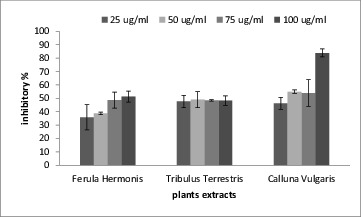
The percentage of inhibition in acetylcholine esterase activity in the presence of the tested plant extracts.

Oxidative stress has long been thought to play a major role in the pathogenesis of AD. Several protective agents such as anti-oxidants, anti-inflammatory drugs, cholinergic agents, estrogens, neurotrophic factors and calcium ion channel antagonists have been proposed for prevention and treatment of AD, but none of them has proved to have a definitive therapeutic effect. Flavonoids are well-known natural compounds possessing a wide range of pharmacological properties related to AD, such as neuroprotective effect, AChE and Aβ fibril formation inhibitory activities, H_2_O_2_-induced ROS formation reduction effect and so on.[36] The study of Jung and Park [37] reported that tiliroside, quercetin, quercitrin and 3-methoxy quercetin which were isolated from the ethyl acetate extracts of the whole plants of *Agrimonia pilosa ledeb* have significant anticholinesterase activity and quercetin was twice as active against AChE than dehydroevodiamine (DHED), which showed more *in vivo* anti-amnesic activity than the clinically useful tacrine. Thus, quercetin or its derivatives might have a significant therapeutic potential for treatment of AD. In agreement with these findings, our results showed that the tested three plant extracts had inhibitory effect toward AChE. The inhibitory effects were concentration dependent as they increased as the extract concentration increased. The most potent inhibitor for AChE was the extract from *C. vulgaris* because it had the lowest IC_50_ (40.8 ± 2.3 μg/mL) as shown in Figure 1 and Table 3.

**Table 3.** IC_50_ of the tested extracts toward AChE and glucosidase.

	IC_50_ (ug/mL)	
Plant extracts	AChE	α-glucosidase
*Calluna vulgaris*	40.8 ± 2.3a	14 ± 1.7b
*Ferula hermonis*	90.9 ± 5.9b	13 ± 1.4b
*Tribulus terrestris*	492.3 ± 12.7c	10.5 ± 0.9a

Within the column, means with different letters (a, b, c, or d ) were significantly different at *p* < 0.05. Mean with letter (a) was significantly the lowest value while mean with the letter (d) was significantly the highest value. If two or three groups have the same letters that means there is no significant difference detected at *p* < 0.05.

Furthermore, type 2 diabetes is an endocrine disease, which accounts for 9% of deaths worldwide. Oral therapy for type 2 diabetes aims to establish normoglycaemia in order that late diabetes complications are prevented.

Among glucose-lowering medications, α-glucosidase inhibitors which delay the absorption of ingested carbohydrates, reduce the post-prandial glucose and insulin peaks.[38] Acarbose represents a pharmacological approach to achieve the metabolic benefits of a slower carbohydrate absorption in diabetes, by acting as a potent competitive inhibitor of intestinal α-glucosidases. Acarbose molecules attach to the carbohydrate binding sites of α-glucosidases, with a much higher affinity constant than the normal substrate.[39] However, the conversion of oligosaccharides to monosaccharides is only delayed rather than completely blocked due to the reversible nature of the inhibitor–enzyme interactions.

**Figure 2.**
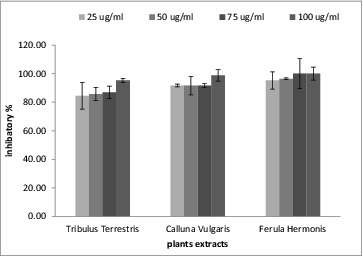
The percentage of inhibition in α-glucosidase activity in the presence of the tested plant extracts.

The three plant extracts had an inhibitory effect towards α-glucosidase, as shown in Figure 2. These inhibitory effects were concentration dependent as they increased as the extract concentration increased. The most potent inhibitor for α-glucosidase was the extract from *T. terrestris* because it had the lowest IC_50_ (10.5 ± 0.9 μg/mL), as shown in Table 3. In comparison to acarbose inhibited the α-glucosidase activity with an IC_50_ value estimated at 765 μg/mL, while the IC_50_ values of the tested extracts ranged from 10.5 to 14 μg/mL, indicative for their very potent α-glucosidase inhibitory properties. The nature of some extract constituents (phenolics, flavonoids and their glycosides) is in accordance with previously reported works that show them to be effective inhibitors of α-glucosidases.[38] Polyphenolic compounds in plants have long been recognized to inhibit the activities of digestive enzymes because of their ability to bind with proteins. Various *in vitro* assays have shown that many plant polyphenols possess carbohydrate hydrolysing enzyme inhibitory activities.[38] Since these molecules exert anti-oxidant effect, it is likely that anti-oxidant and α-glucosidase inhibitory properties should be due to their polyphenolic content.

**Figure 3.**
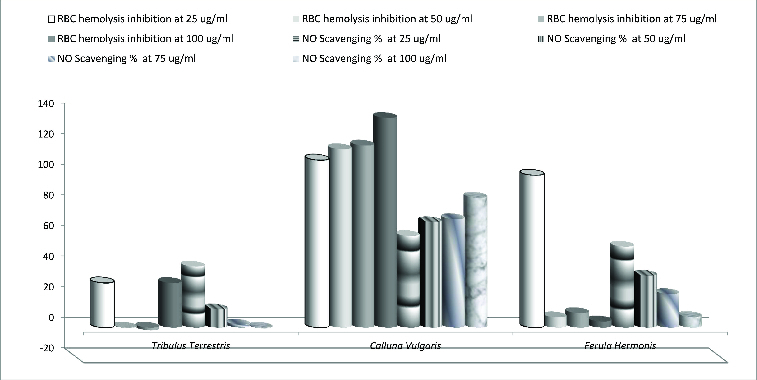
Anti-inflammatory effect of the tested extracts.

The lysosomal enzymes released during inflammation produce a variety of disorders. The extracellular activity of these enzymes is believed to be related to acute or chronic inflammation. The NSAIDs act either by inhibiting these lysosomal enzymes or by stabilizing the lysosomal membrane. In addition to ROS, nitric oxide is also implicated in inflammation, cancer and other pathological conditions.[40] The *in vitro* anti-inflammatory activity of the tested extracts was assessed by HRBC membrane stabilizing method and nitric-oxide-scavenging assays (Figure 3). The extracts from *T. terrestris* and *F. hermonis* at concentration of 25 μg/mL (the low concentration used in this study) had the highest anti-inflammatory effect as the HRBC membrane stability (%) and NO scavenging (%) showed their highest values among the various concentrations of other plant extracts. Otherwise, the *C. vulgaris* extracts showed a dose-dependent anti-inflammatory effect as when the extract concentration was increased, the anti-inflammatory action was also progressively increased. Since HRBC membranes are similar to lysosomal membranes in terms of components, the prevention of hypotonicity that induced HRBC membrane lysis is considered to be a measure of anti-inflammatory activity of the tested extracts.[41]

Our results showed that the tested plant extracts exhibited an anti-inflammatory effect. The results from this study exemplified that the observed significant anti-inflammatory activity of the plant extracts was due to the presence of the above-mentioned constituents. Earlier studies also reported that when tannins, flavonoids, triterpenoids and phenolic compounds [42] are present in extracts, these determined an anti-inflammatory activity. However, it should be noted that despite the shown potent anti-inflammatory effect at low concentration, the extracts from *F. hermonis* and *T. terrestris* exhibit a toxic effect applied in higher concentrations as it was documented. This indicated either that the compounds had cytotoxic properties or that they could increase the production of inflammatory molecules which lead to necrosis. In agreement with this hypothesis, El-Thaher et al. [43] reported that chronic administration of *F. hermonis* exerted unwanted and toxic effects such as decrease in total body weight, hepatomegaly and atrophy of testis. Hussain et al. [44] indicated that the plant extract could lead to cell death through inhibition of chromosomal protein synthesis that in turn might cause a weakening of chromosomal backbone and subsequent chromosomal aberrations.

The results given in Table 4 show that ethanolic plant extract of *C. vulgaris* had a significant effect against most pathogenic bacteria (*Ag. tumefaciens*, *Erwinia* sp., *K. pneumoniae* and *P. aeruginosa*) in comparison to the other extracts from *Ferula* sp. or *Tribulus* sp. Extracts from *Tribulus* sp. had an effect only against *Ag. tumefaciens* and *K. pneumoniae*. On the other hand, the extract from *Ferula* sp. affected only *Ag. tumefaciens*. In agreement with our results, it is reported that *F. hermonis* extracts showed antimicrobial activity against several microorganisms such as Gram positive (*S. aureus*, ***Bacillus*** *subtilis*), as well as the *Mycobacterium* strains *M. bovis* and *M. tuberculosis* H37Rv^21^. *Tribulus terrestris* extracts showed good anti-bacterial activity against Gram positive and Gram negative bacteria. Finally, heather contains many antimicrobial compounds and even honey made from heather flowers has been shown to have high anti-bacterial activity compared to many other types of honey.[45]

**Table 4.** Antimicrobial activity of the three plant extracts on selected pathogenic bacteria.

	Size of inhibition zone (cm)			
	Different concentrations of *Calluna vulgari*s extract			
Bacterial pathogens	0.23 g/mL	0.15 g/mL	0.08 g/mL	0.04 g/mL
*Ag. tumefaciens*	4	3.5	3	–
*Erwinia* sp.	2.5	2.5	2.3	1.8
*Klebsiella* sp.	3	3	1.3	1.1
*Escherichia coli*	–	–	–	–
*Proteus* sp.	–	–	–	
*Pseudomonas aeruginosa*	2.8	3	2.5	1.2
	Different concentrations of *Tribulus terrestri*s extract			
	0.34 g/mL	0.23 g/mL	0.14 g/mL	0.06 g/mL
*Ag. tumefaciens*	1.3	1	–	–
*Erwinia* sp.	–	–	–	–
*Klebsiella* sp.	1.9	1.8	1.7	1.2
*Escherichia coli*	–	–	–	–
*Proteus* sp.	–	–	–	–
*Pseudomonas aeruginosa*	–	–	–	–
	Different concentrations of *Ferula hermonis* extract			
	0.23g/mL	0.15 g/mL	0.08 g/mL	0.04 g/mL
*Ag. tumefaciens*	3.5	3.5	–	–
*Erwinia* sp.	–	–	–	–
*Klebsiella* sp.	–	–	–	–
*Escherichia coli*	–	–	–	–
*Proteus* sp.	–	–	–	–
*Pseudomonas aeruginosa*	–	–	–	–

## Conclusions

All tested extracts could be promising sources for the treatment of diabetes, AD, infectious diseases and oxidative-stress-related disorders because they are rich in phenols and flavonoids that give anti-oxidant molecules and produce an inhibitory effect against the tested enzymes.

## References

[cit0001] Manley JB, Anastas PT, Berkeley WC (2008). Frontiers in green chemistry: meeting the grand challenges for sustainability in R&D and manufacturing. J Cleaner Production.

[cit0002] Saaby L, Rasmussen HB, Jäger AK (2009). MAO-A inhibitory activity of quercetin from *Calluna vulgaris* (L.) Hull. J Ethnopharmacol.

[cit0003] Kumarasamy Y, Philip JC, Marcel J, Lutfun N, Satyajit DS (2002). Screening seeds of Scottish plants for antibacterial activity. J Ethnopharmacol.

[cit0004] Dussossoy E, Brat P, Bony E, Boudard F, Poucheret P, Mertz C, Giaimis J, Michel A (2001). Characterization, anti-oxidative and anti-inflammatory effects of Costa Rican noni juice (*Morinda citrifolia* L.). J Ethnopharmacol.

[cit0005] Ibraheim ZZ, Abdel, -Mageed WM, Huanqin D, Guo H, Zhang L, Jaspars M (2012). Antimicrobial antioxidant daucane sesquiterpenes from *Ferula hermonis* Boiss. Phytotherapy Res.

[cit0006] Hussain AA, Mohammed AA, Ibrahim HH, Abbas AH (2009). Study the biological activities of *Tribulus terrestris* extracts. World Acad Sci Eng Technol.

[cit0007] Williamson EM. (2002). Major herbs of Ayurveda.

[cit0008] Mohamed AA, Khalil AA, El-Beltagi H (2010). Antioxidant and antimicrobial properties of kaff maryam (*Anastatica hierochuntica*) and doum palm (*Hyphaene thebaica*). Grasas Y Aceites.

[cit0009] Padmanabhan P, Jangle SN (2012). Evaluation of in-vitro anti-inflammatory activity of herbal preparation, a combination of four medicinal plants. Int J Basic Appl Med Sci.

[cit0010] Ferreira A, Proença C, Serralheiro ML, Araújo ME (2006). The *in vitro* screening for acetylcholinesterase inhibition and antioxidant activity of medicinal plants from Portugal. J Ethnopharmacol.

[cit0011] Fahimeh M, Asghari B, Saeidnia S, Ajani Y, Mirjani M, Malmir M, Bazaz RD, Hadjiakhoondi A, Salehi P, Hamburger M, Yassa N (2012). *In vitro* α-glucosidase inhibitory activity of phenolic constituents from aerial parts of *Polygonum hyrcanicum*. DARU J Pharm Sci.

[cit0012] Cowan MM (1999). Plant products as antimicrobial agents. Clin Microbiol Rev.

[cit0013] Namita P, Mukesh R (2012). Medicinal plants used as antimicrobial agents: a review. Int Res J Pharm.

[cit0014] Edeoga HO, Okwu DE, Mbaebie BO (2005). Phytochemical constituents of some Nigerian medicinal plants. Afr J Biotechnol.

[cit0015] Shamsa F, Monsef H, Ghamooshi R, Verdian-rizi M (2008). Spectrophotometric determination of total alkaloids in some Iranian medicinal plants. Thai J Pharm Sci.

[cit0016] Hiai S, Oura H, Nakajima T (1976). Color reaction of some sapogenins and saponins with vanillin and sulfuric acid. Planta Med.

[cit0017] Slinkard K, Singleton VL (1977). Total phenol analysis: automation and comparison with manual methods. Am J Enol Viticulture.

[cit0018] Chang CC, Yang MH, Wen HM, Chern JC (2002). Estimation of total flavonoid content in propolis by two complementary colorimetric methods. J Food Drug Anal.

[cit0019] Lowry OH, Rosebrough NJ, Farr AL, Randall RJ (1951). Protein Measurement with the Folin phenol reagent. J Biol Chem.

[cit0020] Jones DL, Owen AG, Farrar JF (2002). Simple method to enable the high resolution determination of total free amino acids in soil solutions and soil extracts. Soil Biol Biochem.

[cit0021] Zollner N, Kirsch K (1962). Microdetermination of lipids by the sulphophosphovanillin reaction. Z Ges Exp Med.

[cit0022] Hedge JE, Hofreiter BT, Whistler RL, Miller JN, Sadasivam S, Manickam A (1982). Carbohydrate chemistry. Biochemical methods for agriculture sciences.

[cit0023] El Sayed M, Ghareeb D, Sarhan E, Khalil A (2011). Therapeutic bio-screening of the bioactive ingredients of *Berberis vulgaris*. FPSB.

[cit0024] Ebrahimzadeh MA, Bahramian F (2009). Antioxidant activity of *Crataegus pentaegyna* subsp. elburensis fruits extracts used in traditional medicine in Iran. Pakistan J Biol Sci.

[cit0025] Anandarajagopal K, Sunilson J, Ajaykumar TV, Ananth R, Kamal S (2013). *In-vitro* anti-inflammatory evaluation of crude *Bombax ceiba* extracts. Eur J Med Plants.

[cit0026] Ebrahimzadeh MA, Nabavi SF, Nabavi SM (2009). Antioxidant activities of methanol extract of *Sambucus ebulus* L. flower. Pakistan J Biol Sci.

[cit0027] Ellman GL, Courtney KD, Featherstone RM (1961). A new and rapid colorimetric determination of acetylcholinesterase activity. Biochem Pharmacol.

[cit0028] Han W, Srinivasan R (1969). Purification and characterization of β-glucosidase of *Alcaligenes faecalis*. J Bacteriol.

[cit0029] Senthil KS, Kamaraj M (2011). Antimicrobial activity of *Cucumis anguria* L by agar well diffusion method. Bot Res Int.

[cit0030] Orhan I, Kupeli E, Terzioglu S, Yesilada E (2007). Bioassay-guided isolation of kaempferol-3-O-β-d-galactoside with anti-inflammatory and antinociceptive activity from the aerial part of *Calluna vulgaris* L. J Ethnopharmacol.

[cit0031] List of medicinal plants and herbs: heather tea. http:/www.liveandfeel.com/medicinalplants.html.

[cit0032] Allouh MZ (2011). Effect of *Ferula hermonis* root extract on rat skeletal muscle adaptation to exercise. Exp Biol Med.

[cit0033] Ebrahimzadeh M, Nabavi S, Nabavi S, Bahramian F, Bekhradnia A (2010). Antioxidant and free radical scavenging activity of H. officinalis L. var. angustifolius, V. odorata, B. hyrcana and C. speciosum. Pakistan J Pharm Sci.

[cit0034] Ebrahimzadeh MA, Pourmorad F, Hafezi S (2009). Antioxidant activities of Iranian corn silk. Turkish J Biol.

[cit0035] Filip GA, Postescu ID, Tatomir C, Muresan A, Clichici S (2012). *Calluna vulgaris* extract modulates NF-κB/ERK signaling pathway and matrix metalloproteinase expression in SKH-1 hairless mice skin exposed to ultraviolet B irradiation. J Physiol Pharmacol.

[cit0036] Shi C, Liu J, Wu F, Yew DT (2010). *Ginkgo biloba* extract in Alzheimer's disease: from action mechanisms to medical practice. Int J Mol Sci.

[cit0037] Jung M, Park M (2007). Acetylcholinesterase inhibition by flavonoids from *Agrimonia pilosa*. Molecules.

[cit0038] Bothon F, Debiton E, Avlessi F, Forestier C, Teulade J, Sohounhloue D (2013). *In vitro* biological effects of two anti-diabetic medicinal plants used in Benin as folk medicine. BMC Complement Altern Med.

[cit0039] Salvatore T, Giugliano D (1996). Pharmacokinetic-pharmacodynamic relationships of acarbose. Clin Pharmacokinet.

[cit0040] Nabavi SM, Ebrahimzadeh MA, Nabavi SF, Hamidinia A, Bekhradnia AR (2008). Determination of antioxidant activity, phenol and flavonoids content of Parrotia *persica* Mey. Pharmacologyonline.

[cit0041] Rajendran V, Lakshmi KS (2008). *In vitro* and *In vivo* anti-inflammatory activity of leaves *of Symplocos cochinchnensis* (Lour) *Moore ssp laurina*. Bangladesh J Pharmacol.

[cit0042] Huang YC, Hwang TL, Chang CS, Yang YL, Shen CN, Liao WY, Chen S, Liaw C (2009). Anti-inflammatory flavonoids from the rhizomes of *Helminthostachys zeylanica*. J Nat Prod.

[cit0043] El-Thaher TS, Matalka KZ, Taha HA, Badwan AA (2001). *Ferula harmonis*’ zallouh’ and enhancing erectile function in rats: efficacy and toxicity study. Int J Impotence Res.

[cit0044] Hussain A, Mohammed A, Ibrahim H, Abbas A (2009). Study the biological activities of *Tribulus terrestris* extracts. World Acad Sci Eng Technol.

[cit0045] Allen KL, Molan PC, Reid GM (1991). A survey of the antibacterial activity of some New Zealand honeys. J Pharm Pharmacol.

